# Asthma and the socio-economic reality in Brazil

**DOI:** 10.1186/1939-4551-6-20

**Published:** 2013-11-12

**Authors:** Eduardo Costa, Mauricio Bregman, Denizar V Araujo, Claudia H Costa, Rogerio Rufino

**Affiliations:** 1 State University of Rio de Janeiro, Rio de Janeiro (20551-030), Brazil

**Keywords:** Asthma, Ambulatory care, Obesity, Health profile, Health system

## Abstract

**Background:**

Asthma is a prevalent disease that is considered a health problem worldwide. The aim of this study was to analyze the clinical and socioeconomic characteristics of a cohort of asthmatics receiving specialized outpatient treatment in a tertiary/teaching public hospital in Brazil.

**Methods:**

Persistent asthmatics older than 5 years old were consecutively included. They received clinical treatment at 3- to 4-month intervals and were interviewed 2 times at 6-month intervals over a 12-month observation period. The data were collected directly from the patients or their parents by 2 researchers who did not participate in their clinical care. The primary variables were age, gender, education level, monthly family income, place of residence, number of lost days of school or work, BMI, the severity and control level of asthma, the number of scheduled and non-scheduled visits and hospitalization days and the best peak-flow measurement.

**Results:**

Of the 117 participants, 108 completed the study. Of the participants, 73.8% were women, and 25.0% lived outside the county. Of those who lived within the county, 60.1% lived in areas far from the health care unit. The majority (83.3%) had associated rhinitis, and more than 50.0% were overweight or obese, in whom the prevalence of severe asthma was greater (p = 0.001). The median monthly income was US$ 536.58 and was greater among the patients with controlled asthma (p = 0.005 and p = 0.01 at the start and the end of the study, respectively). In the initial evaluation, 16 participants had severe asthma, and in the final evaluation, 8 out of 21 patients with uncontrolled asthma had improved. Three-quarters of the students and half of the workers had missed days of school or work, respectively. The asthmatic population in this study had medium to low socioeconomic status in Brazil and socioeconomic status was associated with overweigth/obesity and with poor control of asthma.

**Conclusion:**

Asthma has a great impact on absenteeism in Brazil. Lower monthly family income and body weight above the ideal level were associated with greater severity and worse control of asthma.

## Background

Asthma is one of the most prevalent chronic diseases in the world and is considered a public health problem worldwide [1,2]. The prevalence of asthma in developed countries increased 50% per decade in the last 40 years of the 20th century, and approximately 250,000 deaths occur worldwide because of asthma each year [1].

Asthma is often associated with chronic rhinitis, which can be allergic or not. Studies indicate that 75% to 80% of the individuals with asthma have allergic rhinitis, and 40% to 50% of the individuals with allergic rhinitis and eosinophilic non-allergic rhinitis have bronchial hyperresponsiveness (BHR) [2-4]. Conservative estimates suggest that 500 million people have allergic rhinitis and 300 million people have asthma around the world [2-4]. The Pan American Health Organization (PAHO) estimates that there are approximately 15 million asthmatics in Brazil [5].

In Brazil, the data from the International Study of Asthma and Allergies in Childhood (ISAAC), conducted in various capital cities, showed that the average prevalence rates of allergic rhinitis were 12.6% and 14.6% in children and adolescents, respectively, and the respective prevalence rates of active asthma were 24.3% and 19% [6]. The ISAAC data obtained in cities in the state of Rio de Janeiro showed that the prevalence of active asthma varied from 13-17% in adolescents [7,8]. There are no recent data on the prevalence rates of rhinitis and asthma in the adult Brazilian population. It is estimated that rhinitis affects 20% or more of the general Brazilian population and asthma may affect up to 10%, which means that 19 to 20 million people may have asthma [9]. Although the number of hospitalizations due to asthma in the Brazilian public health system decreased between 2000 and 2011, it remains one of the main causes of hospitalization and imposes a large social and economic burden on the country [9]. Comparing population-based data on adults in Southern Brazil from 2000 to 2010, Fiori and collaborators found that the prevalence rates of asthma were 4.2% and 5.2%, respectively [10].

The Brazilian health system executes the principles defined in the Brazilian Constitution of 1988, which characterized health as a “citizenship right and a duty of the State”. According to the guidelines defined by Law no. 8080/1990 [11], the health system is characterized by universal access and a regional hierarchy of services. Although there have been several programs to organize asthma care at different levels of complexity in the Unified Health System [*Sistema Único de Saúde* – SUS], even large urban centers have not been able to guarantee access for the entire population to the most appropriate level of treatment for the severity of their disease. This obstacle prevents optimal control of asthma and thus prevents reducing its associated morbidity and mortality.

Broad knowledge on the various aspects of chronic diseases, including their clinical and socio-demographic characteristics and the care-seeking behavior of the individuals affected who require health services at different levels of complexity, can optimize the allocation of resources for primary, secondary and tertiary prevention in the Brazilian health system.

The objective of this study was to describe the characteristics of a cohort of patients with persistent asthma who sought specialized treatment in a secondary/tertiary ambulatory care center linked to a university hospital in a large city in Brazil. We analyzed socio-demographic and clinical variables as potential indicators of patient care-seeking behavior at the local health care facilities.

## Methods

Patients older than 5 years of age with a diagnosis of persistent asthma based on previously established consensus criteria [2,9] and who had been receiving treatment for at least 3 months in a specialized ambulatory care center (Allergy-Immunology and Pneumology-Phthisiology Services) in a university hospital in Rio de Janeiro city were consecutively enrolled in the study from April to September 2011. In both services, ambulatory care is predominantly performed by physicians in a residency program. In 2011, 326 patients with asthma were monitored in these services. All of the patients underwent routine visits at 3- to 4-month intervals and were interviewed twice during this period after a 6-month interval. All interviews were taken in the same day of a scheduled clinical visit. The last interview was performed in March 2012. The data were collected using a tool developed by the authors, which was pre-tested with 30 patients before the beginning of the study. Two interviewers (the first and second authors of the present study) who did not participate in the clinical care of the patients received training and performed the data collection on both occasions. In interviews with individuals younger than 18 years of age, all of the questions were answered by a responsible adult, and individuals older than 14 years of age offered supplementary answers when necessary.

The following variables were collected:

–socio-demographic variables: age, gender, education level, monthly family income, the neighborhood where the patients were residing on the day of the interview and the number of days the patient was absent from school or work in the 3 months preceding the interview (to avoid recall bias);

–clinical variables: BMI on the day of the interview, the severity and control of the patient’s asthma in the month preceding the interview, the number of scheduled appointments the patient attended and the number of days of hospitalization in the 6 months preceding the interview and the number of unscheduled/emergency visits in the 3 months preceding the interview (to avoid recall bias);

–functional variable: the highest of 3 peak expiratory flow (PEF) readings on the day of the interview.

The severity (intermittent, mild persistent, moderate persistent and severe persistent) and the control of the patient’s asthma (controlled, partially controlled and uncontrolled) were assessed by the attending physician at the time of the interview using clinical data from the past 4 weeks in accordance with international guidelines [2,9]. The treatment regimen was only changed during the study by the attending physician if a change was indicated according to the same guidelines [2,9]; the interviewers who collected the data after the medical care was provided had no influence.

Overweight and obesity were defined as BMI ≥ 25 and BMI ≥ 30, respectively, for individuals older than 18 years and as weight-for-the age > Z score + 1 SD and weight-for-age > Z score + 2 SD, respectively, for those less than 18 years of age [12]. Annual estimative of school and work losses, as well of unscheduled/emergency visits had made by doubling results of these variables, because they were collected regarding the three previous months.

The patients who dropped out of treatment (i.e., had no return visits for > 4 months) and the patients with chronic cardiopulmonary disease that could cause respiratory symptoms similar to asthma and influence the use of treatment resources were excluded from the study. Before the data collection, the patients (or the guardians if the patient was under 18 years old) signed the free informed consent form after being informed about the study. The study was approved by the local Research Ethics Committee (REC). The project was registered with the Brazilian National Research Ethics Committee [*Comissão Nacional de Ética em Pesquisa* - CONEP] under number FR413262 and approved by the local REC on 05/03/2011.

All of the data were entered in spreadsheets (MS Office/Excel 2010, Microsoft Co., CA, USA) by the first author of the present study. GraphPad Prism version 6.0 (GraphPad Software Inc., La Jolla, CA, USA) was used for statistical analysis. The chi-square test (with Fisher’s correction when necessary) and the Mann–Whitney test were used to compare the differences between categorical variables and the differences between continuous unpaired variables, respectively. The paired t-test and Wilcoxon’s test were used to compare continuous paired variables. A significance level of 5% was used.

## Results and discussion

During the study period, 117 patients were enrolled. Nine patients did not complete the study (7.7% loss): 1 patient died of a cause unrelated to asthma, 1 patient asked to be discharged and 7 patients dropped out of treatment before the second interview. The final study population (108 patients, representing 33.12% of the asthmatic patients in treatment in the two Services) included 80 patients who were followed at the Allergy/Immunology Service and 28 patients from the Pneumology Service. Of the participants, 79 were women (73.8%) and 29 were men (27.1%). This female gender predominance was absent among patients under 20 years-old. Most of the patients (75.0%) resided in Rio de Janeiro city; however, 60.1% of these residents lived in remote areas (defined as more than 10 km distant from the health care unit).

The median monthly family income was R$ 1,100.00 (equivalent to US$ 536.58; IQR25-75 = 348.78-975.61), which falls into the economic classes C1 and C2 [13], considered the lowest stratum of medium economic class in Brazil. Among our patients the primary education level predominates (n = 76/70.4%), 25 (23.1%) have secondary level and only 7 (6.5%) have tertiary or post-graduate educational level, with median monthly family income of US$ 487.80 (IQR 25–75 = 290.24-709.76), US$ 780.49 (IQR 25–75 = 414.63-975.61) and US$ 1,365.85 (IQR 25–75 = 1268.29-1951.22), respectively. Distributions and medians of gender, local of residence, occupational status, educational level, age, duration of asthma and of rhinitis, peak-flow measurements and monthly income are shown in Table 1.

**Table 1:** General characteristics of the patients

**Source – N (%)**	Allergy-Immunology	80 (74.07)
	Pneumology	28 (25.93)
**Gender – N (%)**	Male	29 (26.86)
	Female	79 (73.14)
**Occupation – N (%)**	Student	24 (22.22)
	Employee	33 (30.55)
	Housekeeper	25 (23.15)
	Retired / pensioner	23 (21.30)
	Unemployed	3 (2.78)
**Residence – N (%)**	Rio de Janeiro city	81 (75.00)
	Rio de Janeiro city but in remote areas	65 (60.19)
	> 10 km from the health care units	
	Other cities	27 (25.00)
**Age (years)**	Median (percentile 25-75)	49.50 (27.75 – 60.00)
**Duration of asthma (years)**	Median (percentile 25-75)	18.00 (9.50 – 33.00)
**Duration of rhinitis (years)**	Median (percentile 25-75)	19.00 (8.00 – 30.75)
**Monthly family income (U$)**	Median (percentile 25-75)	558.13 (356.97 – 930.23)
**Peak flow meter**	Median (percentile 25%-75%)	80 L/m (65 - 89)

Ninety patients (83.3%) had chronic rhinitis associated with asthma. The proportion of asthma cases associated with rhinitis was higher among the patients from the Allergy-Immunology Service (p = 0.0001), whereas the patients from the Pneumology Service were older, had asthma for a longer duration, experienced more severe asthma and had a greater prevalence of overweight/obesity (p = 0.02, p = 0.04, p = 0.01 and p = 0.01, respectively).

Sixty-seven patients (62.0% of the total) had another medical comorbidity: 41 out of them (61.2% of patients with comorbidities) had systemic arterial hypertension (SAH), none were using beta-blockers, 11 (16.4%) had diabetes mellitus (DM), and 11 (16.4%) had degenerative joint disease. Other less common comorbidities included thyroid disease, depression, dyslipidemia and gastro-esophageal reflux. Table 2 shows distribution of gender, monthly family income, weight status and presence of self-referred comorbidities by age ranges.

**Table 2:** Gender, family income, weight status and self-referred comorbidities distribution by age ranges

**Characteristics by age ranges**				
**Age range(years)**	**Gender N (%)**	**Monthly income (US$)**	**Weight status N (%)**	**Self-referredco-morbidities N (%)**
		**Median (IQR25-75)**		
< 20	Male = 12 (57)	439.02	Obese = 0 (0.0)	Yes = 06 (28.5)
Total=21	Female = 09 (43)	(292.68-731.70)	Overweight = 02 (9.5)	No = 15 (71.5)
			Normal = 19 (90.5)	
20 – 39	Male = 03 (17.7)	975.61	Obese = 06 (35.3)	Yes = 08 (47.0)
Total=17	Female = 14 (82.3)	(414.64-1,317.07)	Overweight = 06 (35.3)	No = 09 (53.0)
			Normal = 05 (29.4)	
40 – 59	Male = 08 (18.2)	536.58	Obese = 14 (31.8)	Yes = 31 (70.5)
Total=44	Female = 36 (81,8)	(382.97-975.61)	Overweight = 16 (36.4)	No = 13 (29.5)
			Normal = 14 (31.8)	
> 60	Male = 05 (19.2)	534.14	Obese = 9 (34.6)	Yes = 22 (84.6)
Total=26	Female = 21 (80.8)	(270.14-780.48)	Overweight = 11 (42.3)	No = 04 (0.0)
			Normal = 06 (23,1)	

**Figure 1:**
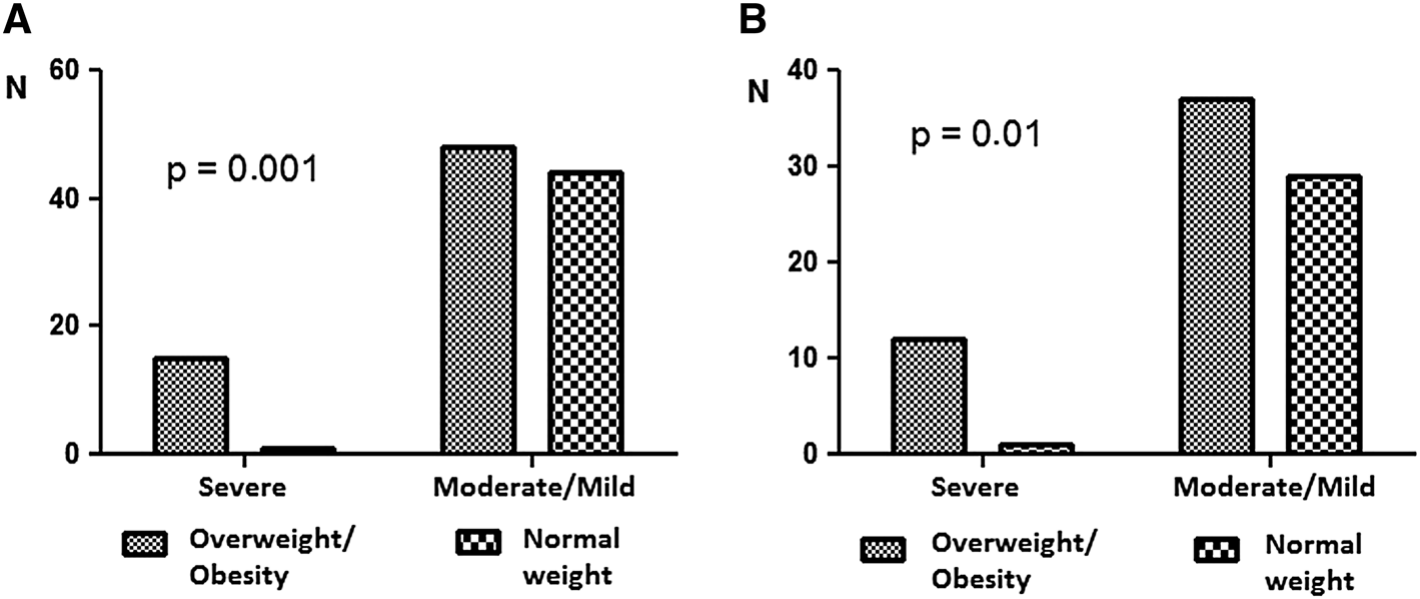
Weight and asthma severity in first evaluation: (A) in whole population; (B) in women.

Of the patients whose body weight was above the healthy weight range (n = 64 / 59.2% of the total), 35 (32.4%) were overweight and 29 (26.8%) were obese. None of children and teenagers were obese and only 2 (9.5%) were overweight, while obesity and overweight predominated among other age ranges (Table 2). The proportion of severe asthmatics was greater than the proportion of mild/moderate asthmatics among the overweight/obese patients compared with the normal weight patients in the start of study (p = 0.001). This difference persisted when we analyzed only women, who represented the majority of the population (p = 0.01) (Figure 1).

**Figure 2:**
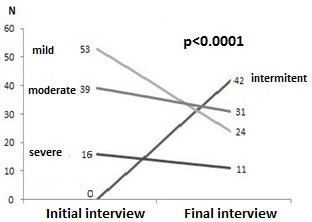
Evolution of asthma severity during the study.

At the beginning of the study, 53 patients (49.08%) were classified as having mild asthma, 39 (36.11%) had moderate asthma and 16 (14.81%) had severe asthma. By the final evaluation, the classification of 42 patients (38.90%) had changed to intermittent asthma, and 8 (38.09%) of the 21 patients who initially had uncontrolled asthma demonstrating the intra-individual variability of the disease and improvements in their control after 6 months of treatment. The average PEF measurements increased significantly during the study (p < 0.0001), and the changes in severity were statistically significant (p < 0.0001) (Figure 2). Futhermore, 12 patients (11.11%) were not taking medication to control their asthma, whereas only 4 patients (3.7%) were not taking it at the time of the second data collection (2 with intermittent/controlled asthma and 2 with moderate persistent asthma; 1 partially controlled and 1 uncontrolled).

The patients attended 444 appointments in the 6-month period before the first interview and 330 appointments in the 6-month period before the second interview (mean 4.11 and 3.05 visits/patient/semester, respectively). There were 108 visits without an appointment (ambulatory care or urgent care/emergency) in the 3 months prior to the first interview and 49 in the 3 months prior to the second interview (mean 1.00 and 0.45 visits/patient/trimester, respectively). The median expenditure with public or private transport to attend these visits was US$ 9.76 per patient (IQR25-75 = 5.37-14.63). Only one and two patients went to visits by foot in the first and second interviews/clinical visits, respectively. In the 6 months preceding the first data collection, 3 patients were hospitalized due to asthma (total of 13 days of hospitalization; mean 4.33 days/patient), and 2 patients (total of 7 days of hospitalization; mean 3.5 days/patient) were hospitalized within the 6 months preceding the second data collection.

**Figure 3:**
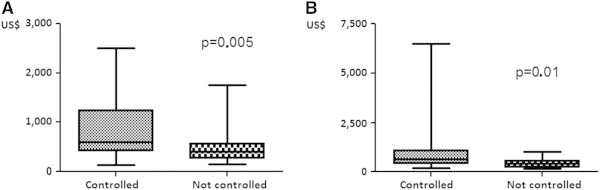
Month family income and asthma control: (A) initial evaluation; (B) Final evaluation (median / percent 25–75 / minimum and maximum).

Monthly family income was lower among the patients with uncontrolled asthma both at the beginning and at the end of the study (p = 0.005 and p = 0.01, respectively; Figure 3). At the beginning of the study, the patients with uncontrolled asthma had a median monthly income of U$ 372.09 (25–75 percentile = U$251.16 – U$534. 88), whereas those with controlled asthma had higher incomes (median = U$ 558.13; 25–75 percentile = U$ 390.69 – U$ 1,162.69). At the end of the study, this difference persisted (uncontrolled asthma: median = U$ 372.09 *versus* controlled asthma: median = U$ 604.65). Regarding severity in the two points of observation, the mean annual family income of patients with severe, moderate, mild and intermittent asthma was U$ 8,115.06, U$ 8,327.64, U$ 10,409.82 and U$ 9,563.76, respectively. Patients with severe and moderate asthma reduced their mean family incomes during the study, whereas mild asthmatics did not. Estimative of annual costs of asthma treatment per patient showed that severe ones expend 12% of family annual income, whereas the moderate asthmatics expend 4.8%, mild patients 3.6% and intermittent ones 3.7% of annual income treating their asthma.

None of the 53 patients with controlled asthma were smokers, but 3 patients with partially controlled or uncontrolled disease were still smoking. Three other patients whom live together with smokers had their asthma each one controlled, partially controlled and not controlled.

**Figure 4:**
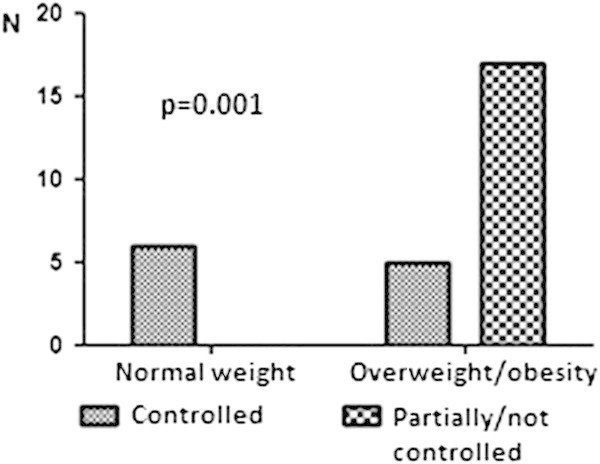
Weight and asthma control among Pulmonology Section’s patients.

At the end of data collection, the difference between asthma control of the patients in the Allergy/Immunology Service compared with patients in the Pneumology Service didn’t reach statistical significance at 5% level (p = 0.08), however we don’t know if it would be different with a greater sample. Of the patients treated at the Pneumology Service (who had more severe asthma and a high prevalence of overweight/obesity), the proportion of patients with partially controlled or uncontrolled asthma was higher among the overweight/obese patients compared with those of normal weight (p = 0.001; Figure 4). There were no differences in gender distribution (p = 0.62) or the monthly family income (p = 0.39) between patients from the two Services.

Eighteen (75.0%) of the 24 students missed school because of asthma, and 13 patients missed work because of their illness (38.3% of employees outside home). Including the 4 employees who were absent from work to take care of their children with asthma, a total of 17 employees (51.5% of the individuals working outside the home) missed work because of asthma in the 3-month periods prior to each interview. The participants missed an estimated total of 163 school days/year and 164 working days/year, or an average of 6.79 days/student/year and 9.64 days/employee/year.

In the first assessment, 4 patients were not working and collecting sickness benefits, including 1 with severe uncontrolled asthma and 3 patients with moderate disease. In the second evaluation, 2 of these patients had retired because of disability related to their asthma, one remained out of work, another one lost her job, and another patient (with partially controlled moderate persistent asthma) had also stopped working and was collecting sickness benefits.

Few published studies with different main objectives have described the profile of asthmatic patients receiving specialized monitoring in medium to high complexity hospitals. It is estimated in published guidelines that 60.0% of asthma cases are intermittent or mild persistent, 25.0% to 30.0% are moderate, and only 5.0% to 10.0% are severe [2,9]. This minority of severe asthmatics utilizes the most costly resources (e.g., medication, non-scheduled ambulatory visits, emergency visits and hospitalizations) and account for most of the mortality caused by the disease. These patients usually have the worst asthma control and are most in need of monitoring at secondary and tertiary heath care facilities [2,9,14].

At the beginning of the study, our results showed that nearly 15.0% of the patients had severe asthma, which was higher than the estimated proportion for the general population of asthmatics but still small for a specialized ambulatory care center in a moderate- to high-complexity unit. We observed a large proportion of patients with mild asthma in this population. In part, this result may be related to the large number of patients followed in Allergy/Immunology Services, where allergic asthma is predominant and the proportion of patients with more severe disease is lower than the proportion of adult patients with non-allergic asthma. Part of the demand from patients with less severe disease could be absorbed by units in the basic health care network if all or most of the local network had health care teams properly prepared to address the disease. Asthma care programs already exist in other regions of the country, which may be one of the reasons why fewer patients are admitted with moderate/severe asthma that requires specialized assistance with more diagnostic and therapeutic resources.

In a study with 90 participants that compared the direct costs of treating patients with controlled and uncontrolled asthma (45 per group) in a Brazilian tertiary health care unit, the mean age was similar to the age of our population, but the average monthly family income was lower [15]. Thirty-one patients had mild asthma (34.4%), 41 had moderate asthma (45.5%), and 18 (20.0%) had severe asthma: compared with our population, a slightly higher proportion of patients had severe asthma and a slightly lower proportion had mild asthma in that unit. Even so, we still consider the percentage of patients with mild asthma treated in a university hospital in the largest Brazilian city to be high, suggesting that the city experiences difficulties similar to ours in distributing the demand from asthmatic patients across the hierarchy of public health care units.

The data from the second evaluation of our patients show that a large proportion of the patients were able to control and/or reduce the severity of their asthma after 6 months. Some of these patients should be referred to less complex units close to their homes, providing greater comfort to the patients and opening up spaces to admit and treat patients with more severe disease. This issue reflects the difficulties in referring patients between the health care units of varying complexity in the health care system.

Approximately 10.0% of the patients did not achieve total or partial control of the disease, even while undergoing treatment in a university hospital where, theoretically, more comprehensive approaches are applied in accordance with the latest guidelines and the access to many diagnostic resources is unrestricted. Limited access to medications for treatment may have contributed to this problem because most of the patients depended on obtaining free samples or buying medications with their own resources.

A retrospective study performed in a Brazilian university reported results similar to ours in terms of the mean age, the proportion of women and the proportion of patients with severe asthma among the patients in treatment [16]. Based on the evaluation of the last prescription in the records, the use of the pharmacological treatment in accordance with the guideline recommendations for the asthma management at that time was low. Among the patients with persistent asthma, a large proportion (71.0%) had no prescription for inhaled corticosteroids. The current guidelines for asthma management clearly state that continuous use of inhaled corticosteroids, tailored to the severity level and control of the disease, is the most effective strategy for reducing morbidity and mortality from persistent asthma [2,9]. The cited study, unlike ours, included patients treated in other services that address asthma but were not specialized in Allergy or Pulmonology, which may have contributed to the undesirable result. This study suggests that even in a university hospital in a large Brazilian city, teams that do not specialize in respiratory diseases (pediatricians, internists and general practitioners) are not properly applying the recommended treatment guidelines for the disease.

Exacerbating the problem of inappropriate prescription practices, there are high rates of non-adherence and inadequate adherence to the treatment regimen (potentially greater than 70%), and the dropout rate from control medications can reach 92.0% after 1 year [17-19]. Our population has a low to medium socio-economic profile, with access to urban transport and medication. Moreover, in our institution, a university health unity, is admired for most of people and has a good reputation in the city. These facts can helped us to reach this low level of losses during the study (only 7 out of 117 allocated patients dropped out of treatment during the 12 months of observation).

In another study that retrospectively analyzed 434 asthmatic children and adolescents included in the assistance program in Brazilian primary health care units between 1988 and 1993 [20], more than 50.0% dropped out during the monitoring phase, predominantly in the first 6 months of treatment. Among those who continued with the monitoring program, the asthma assistance program in the primary health care unit achieved successes in terms of clinical improvement and greater adherence to drug treatments [21].

In our population of patients with persistent asthma, 11 (10.1%) were not taking continued inhaled corticosteroid at the beginning of the study (2 uncontrolled, 5 partially controlled and 4 considered controlled). The mean monthly income of them was not significantly different from the rest of patients using inhaled corticosteroids (US$ 595.61/SD = 355.70 *versus* US$ 764.39/SD = 746.34; p = 0.21). The proportion of non users of inhaled corticosteroids dropped to 3.0% (2 patients with uncontrolled and partially controlled asthma, each one) of the 66 patients with persistent asthma at the end of the study. In addition, the number of unscheduled visits to the ambulatory care unit or emergency care decreased throughout the study. The study was observational; there was no active attempt by the researchers to change the patients’ therapy because they had already been receiving treatment for at least 3 months when they joined the study. These data suggest that the local teams are capable of providing competent care in alignment with the current recommendations for drug treatment for asthma [2,9]. We can’t rule out the possibility that patients’ adherence improved when they were informed that they were participating in a longitudinal study after the first data collection that would include a second data collection (Hawthorne effect) and/or that the teams paid more attention to the medications of the participants between the two data collection points because the support staff could identify the patients participating in the study. Furthermore, asthma severity and asthma control naturally vary over time. The first data were collected in the fall and winter, whereas the second collection occurred during the spring and summer, when the weather contributes to better clinical outcomes of asthma in our geographic region.

Our results demonstrate that asthma has a strong impact on school and work attendance because 75.0% of the students missed days of school and 34% of employees missed days of work directly because their asthma. This proportion grows to more than 50% if we also consider adults that missed work to care their children with asthma. Our prevalence of work absenteeism are clearly bigger than published results from a cohort of industry employees aged 16 to 65 years old in Brazil, where the one year prevalence of work days lost to health problems was 13.5% [22]. In addition to causing absences from work when the disease is exacerbated, asthma also causes long temporary absences. In Brazil in 2008, sickness benefits for asthma were provided to 7.5/100,000 employees for a median duration of 49 days (IQR 25–75 = 28–87 days) [23]. In a transversal study on health related work days lost during 30 months among public service workers in Vitória, a medium seaside city in the same region of Rio de Janeiro (southwest of Brazil), respiratory diseases was the first cause of absenteeism with an average of 8.4 days and median of 5 days lost per period of license [24]. In the population that we studied, 5 patients (4.6% of total and 15.1% of employees) with moderate to severe asthma were out of work during the study period, 2 retired due to disability, and 1 lost a job. The monthly income of patients with uncontrolled asthma was lower than the income of those with controlled asthma at both observation points. Despite the small number of patients with uncontrolled disease, and considering that only 5 patients (6.6% of all working aged asthmatics) were out of work or retired due to asthma, our results suggest that lower income can contribute to worse the disease. However, the lower mean monthly family incomes of severe patients compared to moderate and mild ones as well as the reduction in these indexes in severe and moderate patients during the study, but not in mild ones, suggest that the disease can also contribute to lower family income reducing the working capacity of patients or their parents.

As the literature has already described, we noted a frequent association between asthma and chronic rhinitis, which reinforces the need to devote attention to treatment that properly controls this comorbidity in asthmatics [3]. In a regional Brazilian program, adults with moderate to severe asthma were monitored and receive inhaled medication to control their asthma. For 21 months in 2003 and 2004, 269 patients with a median age of 46 years were included in a study of the characteristics and costs of asthma. Rhinitis was present in 72% of the patients, a lower proportion than what we found despite the greater severity of asthma in those patients. Less than half of the patients were working, and the second and third-largest proportions were composed of unemployed people and retirees, respectively. The majority (74%) had a monthly family income less than the national minimum wage (i.e., the population was poorer than the one followed in our study). Nevertheless, with free medication, amelioration of asthma control and a reduction in hospitalizations were achieved [25].

Other clinical comorbidities were also common in our population, especially overweight/obesity and systemic arterial hypertension, which can impair the control of asthma or compete for financial resources used for asthma treatment, respectively. Overweight and obesity were associated with increased asthma severity in all of the patients and with worse control of the disease among the patients with more severe disease, older patients and patients with a longer duration of asthma. None of the asthmatic patients with systemic arterial hypertension were using a beta-blocker, which is known to aggravate asthma.

Another study conducted by the Bahia State Asthma and Allergic Rhinitis Control Program (*Programa de Controle da Asma e Rinite Alérgica na Bahia -* ProAR) in Brazil sought to evaluate the factors associated with severe asthma in the population [26]. Clinical data from 102 asthmatics treated in 2007–2008 were evaluated retrospectively. The mean age was 44.0 years (± 13.6). Only 2.9% of the patients had mild asthma, 30.4% had moderate asthma, and 66.7% had severe asthma, as expected for a specialty service for asthma. In this population, 61.7% of the patients were overweight or obese, a proportion similar to our findings, even though we observed fewer severe cases. There was also a significant association between arterial hypertension and asthma.

The increasing prevalence of overweight/obesity in Western societies has been identified as a factor associated with the increased prevalence of asthma. Data from the USA show that the prevalence rates of asthma and obesity increased to a similar extent between 1980 and 2000 [27]. Data of Brazilian Health Ministry, obtained by a telephone surveillance system in 2011 showed that 64.3% of Brazilian adults had overweight or obesity, a similar result compared with ours, but with a smaller proportion of obese (15.8%) and more overweight (48,5%) [28]. Studies have shown that obesity is associated with an increased risk of asthma symptoms. This association could initiate in early life, being greater in adults than in children and in adult women than in men, but the nature of these potential reciprocal effects still need further investigation [29]. Obesity appears to contribute to reduce responsiveness to medication, worsening control and increasing associated costs of the disease. This could be due to a change in asthma phenotype, particularly evidenced as a less eosinophilic type of airway inflammation with less responsiveness to inhaled conticosteroids, combined to the added effects of changes in lung mechanics [30-32].

These results reinforces that, besides providing free access to control medication, which recently was became available by Brazilian government, the public health system needs to make efforts to provide primary health facilities with interdisciplinary teams prepared to approach the various educational, socioeconomic and clinical aspects of asthma and its comorbidities to medium/lower income population. Focus in drug treatment as defined by international guidelines, continuing clinical and functional monitoring and adequate approaching to rhinitis and obesity are needed.

## Conclusions

The population of individuals with asthma followed in this moderate- to high-complexity health care unit had medium-low socioeconomic status in Brazil, a high prevalence of associated chronic rhinitis and a high prevalence of overweight/obesity. A large proportion of the patients have missed days of school or work for reasons were directly or indirectly related to the disease. Lower monthly family income and body weight above the ideal level were associated with greater severity and worse control of asthma. In Brazil, there are difficulties in building a regional and hierarchical public health system, which may be the consequence of an inadequate supply of services.

## Competing interests

There aren’t any competing interests of the authors in this manuscript.

## Authors’ contributions

All authors read and approved the final manuscript.
